# A Comprehensive Assessment Protocol for Swallowing (CAPS): Paving the Way towards Computer-Aided Dysphagia Screening

**DOI:** 10.3390/ijerph20042998

**Published:** 2023-02-08

**Authors:** Hyo-Jung Lim, Derek Ka-Hei Lai, Bryan Pak-Hei So, Calvin Chi-Kong Yip, Daphne Sze Ki Cheung, James Chung-Wai Cheung, Duo Wai-Chi Wong

**Affiliations:** 1 Department of Biomedical Engineering, Faculty of Engineering, The Hong Kong Polytechnic University, Hong Kong, China; 2 School of Medical and Health Sciences, Tung Wah College, Hong Kong, China; 3 School of Nursing, The Hong Kong Polytechnic University, Hong Kong, China; 4 Research Institute of Smart Ageing, The Hong Kong Polytechnic University, Hong Kong, China

**Keywords:** dysphagia, deglutition disorder, eating disorder, otorhinolaryngology, computer-aided diagnosis, wearable technology

## Abstract

Dysphagia is one of the most common problems among older adults, which might lead to aspiration pneumonia and eventual death. It calls for a feasible, reliable, and standardized screening or assessment method to prompt rehabilitation measures and mitigate the risks of dysphagia complications. Computer-aided screening using wearable technology could be the solution to the problem but is not clinically applicable because of the heterogeneity of assessment protocols. The aim of this paper is to formulate and unify a swallowing assessment protocol, named the Comprehensive Assessment Protocol for Swallowing (CAPS), by integrating existing protocols and standards. The protocol consists of two phases: the pre-test phase and the assessment phase. The pre-testing phase involves applying different texture or thickness levels of food/liquid and determining the required bolus volume for the subsequent assessment. The assessment phase involves dry (saliva) swallowing, wet swallowing of different food/liquid consistencies, and non-swallowing (e.g., yawning, coughing, speaking, etc.). The protocol is designed to train the swallowing/non-swallowing event classification that facilitates future long-term continuous monitoring and paves the way towards continuous dysphagia screening.

## 1. Introduction

### 1.1. Background of Dysphagia

Dysphagia is a medical term used to describe swallowing difficulties [1]. Dysphagic patients may experience pain or difficulty while swallowing, drinking, eating, or regulating their saliva, and/or taking medications. In severe situations, the bolus may enter the airway and lungs, causing aspiration pneumonia, a deadly but clinically asymptomatic condition [2]. The prevalence of dysphagia has been estimated at 25% in the adult population but could be as high as 41%, 45%, and 60%, respectively, for those with post-stroke, institutionalized dementia, and Parkinson’s disease [3,4,5]. About one-third of older adults with dysphagia live alone, and more than two-thirds reside in nursing facilities, which represents an imminent threat to the healthcare system and society [6]. Dysphagia causes other problems in older adults, such as malnutrition and dehydration, which might eventually lead to general health issues or even death [7]. People with dysphagia have a 1.7 times greater mortality risk and spend USD 6000 more on hospitalization per discharge than those without [8]. Additionally, the fear of choking significantly decreases their quality of life and mental well-being [9].

### 1.2. Traditional Swallowing Assessments for Dysphagia

Establishing an effective swallowing assessment is imperative to prompt older adults who are at risk to take rehabilitative measures and to evaluate the effectiveness of the rehabilitative measures. Clinically, the fiber-optic endoscopic evaluation of swallowing (FEES) and the video-fluoroscopic swallowing study (VFSS) are the gold standards for dysphagia diagnosis [10]. The FEES procedure involves passing the endoscopic instrument through the nose to observe the pharynx and larynx when the individual is swallowing saliva with and without food of varying consistencies [10]. VFSS applies the same principles but uses fluoroscopy over the oral cavity, pharynx, and cervical esophagus [10]. However, these methods have several disadvantages. FEES induces pain and discomfort, and VFSS exposes patients to radiation [10]. Moreover, FEES and VFSS are costly and require professionals for their operation, which might not be feasible for community screening.

Non-instrumental swallowing evaluations are routinely conducted and play an important role in bedside dysphagia screening [11]. These clinical examinations include a morphodynamical evaluation of the oral–neck region in addition to some clinical tests, such as normal and pathological reflex, an oral feeding test, water swallowing test, and gustative function test [11,12]. Additionally, clinicians or trained experts may listen to the swallowing sound using a stethoscope to identify abnormality, a technique known as cervical auscultation [13]. However, these processes are subjective, and some require trained personnel or clinicians to conduct. Overall, they have poor sensitivity, reproducibility, and predictive strength [14,15].

There are other drawbacks of the standard instrumental diagnosis and routine screening tools. These tests are typically conducted once at a single timepoint because frequent and continuous assessments are often not feasible or affordable. In fact, the occurrence of dysphagia is a gradual process, along with the deterioration of cognitive functions, especially in the dementia population [16,17]. Continuous monitoring or frequent dysphagia screening is essential in order to mitigate the risk of choking and aspiration [18].

### 1.3. Emerging Computer-Aided Screening Technologies for Dysphagia

Emerging wearable technology might provide the opportunity to enable continuous monitoring. Swallowing difficulties manifest physical characteristics that could be directly or indirectly measurable, including longer swallowing times and delayed pharyngeal initiation accompanied by poor epiglottic inversion, hyolaryngeal movement, and pharynx clearance [17]. Existing wearable devices for swallowing assessment include accelerometers, acoustic sensors (e.g., microphones), an electromyogram (EMG), flexible biosensors using biomaterials, etc., while machine learning, deep learning models, or other algorithms could facilitate the identification of swallowing events [19]. Nevertheless, to distinguish dysphagic and non-dysphagic individuals, the wearable system and models/algorithms should first be able to differentiate swallowing and non-swallowing events correctly, since the measurements would not only be taken during swallowing episodes. Unfortunately, our previous review found that the accuracy of wearable technology in predicting swallowing events was poor and, therefore, insufficient to facilitate further applications for dysphagia screening [19]. This could be due in part to the lack of a comprehensive and standardized assessment protocols for swallowing [19]. Remarkably, the challenges lie in the fact that individuals execute plenty of different otolarynpharyngeal biomotions throughout the day, such as reading, coughing, and throat clearing.

### 1.4. Scope and Objectives

To this end, the objective of this study is to develop a Comprehensive Assessment Protocol for Swallowing (CAPS) that is dedicated but not exclusive to wearable technology with machine learning or other classifier algorithms. The premise of this protocol was built upon an integration of our previous review synthesis [19], the International Dysphagia Diet Standardization Initiative (IDDSI) framework [20], and face validity by our team of biomedical engineers, geriatric nurses, and occupational therapists.

## 2. Methods

### 2.1. Protocol Overview

The protocol consists of two phases: (1) the pre-test phase and (2) the assessment phase. It should be completed in a single visit by trained personnel or clinicians, such as nurses, speech therapists, and occupational therapists, to oversee any risk of choking and other adverse events. The pre-test phase involves wet swallowing tasks to determine the size of bolus intake. The assessment phase involves dry (saliva) swallowing, non-swallowing, and wet swallowing tasks in sequence. Measurements and assessments are carried out in this phase to screen for individuals with potential dysphagia or swallowing abnormalities. The participants can request a break at any time and should be reminded to pause the task and report any discomfort to the observer.

**Figure 1:**
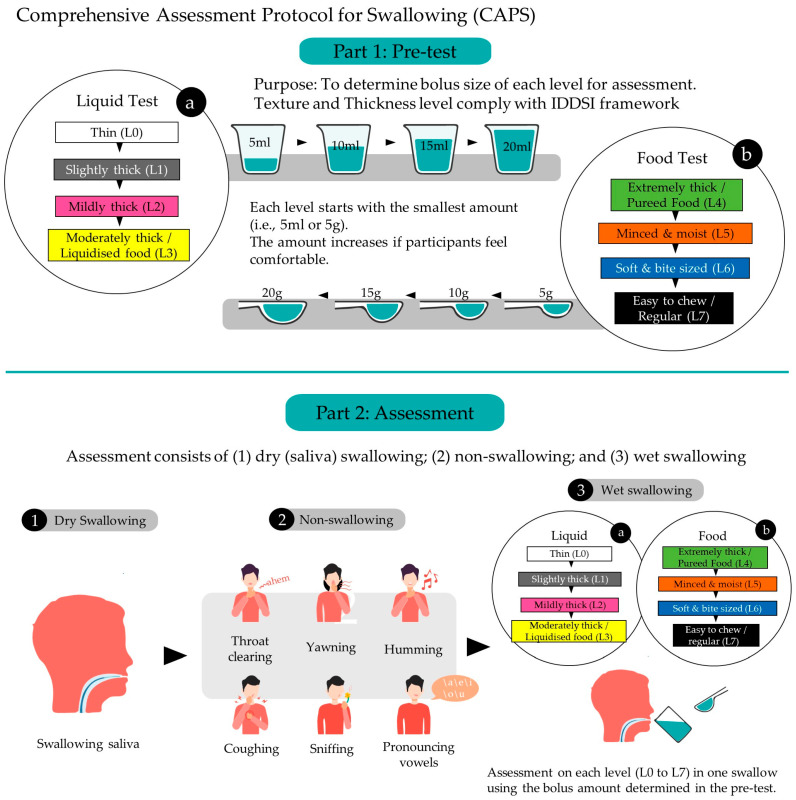
Overall framework and sequence of the Comprehensive Assessment Protocol for Swallowing (CAPS).

**Figure 2:**
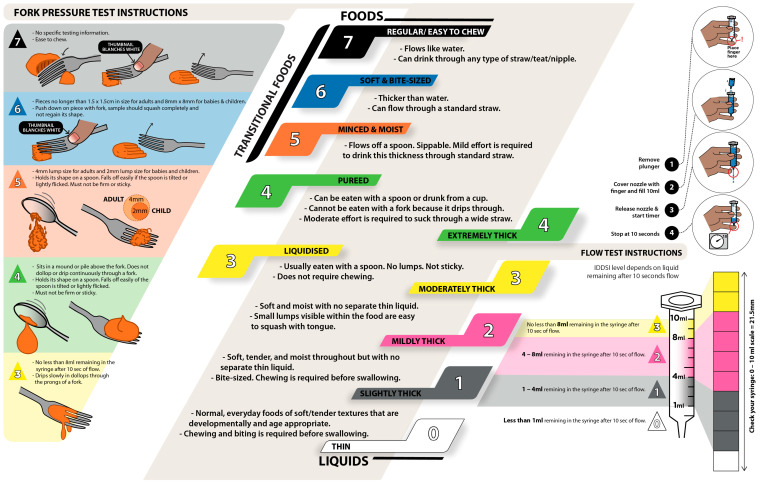
Food and liquid samples prepared according to the fork pressure test and flow test from the International Dysphagia Diet Standardization Initiative (IDDSI) framework (original figure, information acquired from © The International Dysphagia Diet Standardization Initiative 2019, @ https://iddsi.org/framework/Licensed (accessed on 5 February 2023) under the CreativeCommons Attribution Sharealike 4.0 License: https://creativecommons.org/licenses/by-sa/4.0/legalcode (accessed on 5 February 2023)).

The overall framework and sequence of the protocol are illustrated in Figure 1. For both phases, the type and texture of food and liquid samples should be prepared according to the IDDSI framework through the fork pressure test and flow test (Figure 2).

### 2.2. Pre-Test Phase to Determine the Size of Bolus Intake for Assessment

The purpose of the pre-test phase is to determine the appropriate size of bolus intake of foods and liquids for the next assessment phase. Participants are asked to avoid eating and drinking for 2 h before the test. The session starts with the participant sitting in a comfortable position and fully alert. They are then asked to drink 10 mL of water to moisturize their mouth. The pre-test begins with liquid samples and is followed by food samples.

The pre-test phase continues sequentially with wet swallowing tasks based on different textures and thicknesses of liquids and foods/transitional foods, as shown in Table 1. For then liquids, the participant starts with Level 0 (a cup of a thin drink) and then progresses through Level 1, Level 2, and ultimately Level 3 (a cup of a moderately thick drink). For each level, the participant is given 5 mL of sample to ingest in a single swallow. If the participant feels comfortable with the current amount, s/he is given a larger volume to swallow stepwise (from 5 mL, 10 mL, and 15 mL to a maximum of 20 mL) until s/he feels difficulty. The observer can stop the volume increment if s/he believes that the participant is not comfortable or cannot accommodate the sample size. The maximum amount of sample that the participant can swallow comfortably is noted and applied in the assessment phase.

For swallowing tasks based on foods/transitional foods, the procedures are very similar. The participant starts with Level 4 (extremely thick food or pureed food) and then progresses through Level 5, Level 6, and Level 7 (easy-to-chew or regular food). For each level, the participants are given 5 g of sample to consume in a single swallow. S/he then attempts to consume an increasing volume (from 5 g, 10 g, and 15 g to a maximum of 20 g) until s/he notes difficulty. Similarly, the maximum amount of sample that the participant can swallow comfortably is noted and applied in the assessment phase. We propose some examples of food or drink according to the IDDSI levels in Table 1.

### 2.3. Assessment Phase

The assessment phase continues after the pre-test and consists of the following tasks in sequence: (1) dry (saliva) swallowing tasks; (2) non-swallowing tasks; and (3) wet swallowing tasks.

For dry (saliva) swallowing, participants are instructed to gather saliva in their mouths and to swallow once. The non-swallowing tasks involve six maneuvers: throat clearing, yawning, sniffing, coughing, humming, and pronouncing vowels, as shown in Table 2. The sequence of the non-swallowing maneuvers should not be adjusted. To begin the wet swallowing phase, the participant drinks 10 mL of water to moisturize their mouth. Next, they repeat the same protocol as that in the pre-test phase. At this time, s/he only needs to swallow one time for each level at their recorded maximum comfortable swallowing volume.

### 2.4. Evaluation/Assessment Methods

The design of the protocol is intended to be generic for different bedside screening techniques and therefore should accommodate the existing evaluation methods, such as clinical examinations and questionnaires.

Moreover, the protocol design is devoted to a computer-aided dysphagia screening tool using wearable technology and machine learning techniques or other classifier algorithms. In such a case, the signals of sensors corresponding to each swallowing maneuver can either be manually labelled by the professional watching the video recording, or be labelled by asking the participant to press a button or pedal during the swallowing episode [21,22]. The tasks during the assessment phase can be repeated according to the need for data augmentation. In order for the technology to learn the signal abnormality of dysphagia, patients with a diagnosis confirmed by VFSS or FEES can be recruited to perform the swallowing tasks with the wearable technology and compared to those without dysphagia.

## 3. Discussion

The proposed Comprehensive Assessment Protocol for Swallowing (CAPS) accounts for the evaluation of swallowing, dry (saliva) swallowing, and non-swallowing events. It integrates the IDDSI framework and other existing protocols in an attempt to assess possible otolarynpharyngeal biomotions. The significance of this protocol is two-fold. Firstly, it is more comprehensive and precise than the traditional one-step approach, in that it considers a variety of biomotions and tests based on different bolus textures and thicknesses. Secondly, the protocol facilitates the training process of the machine learning model for computer-aided dysphagia screening. Once the model is trained, professionals might not need to perform standalone swallowing assessments using CAPS or other bedside screening tests. Instead, the wearable system can monitor the swallowing process and objectively evaluate the risks continuously, even over a prolonged period of time. It should be noted that this protocol paper proposes tasks for the swallowing assessment but not the instrument of the assessment. Regardless, the accuracy, reliability, and validity of different instruments should be well-evaluated. The diagnosis of dysphagia should also be confirmed by standard instruments (e.g., VFSS and FEES) after screening.

The wet swallowing tasks draw on the IDDSI framework. In the framework, the texture and thickness levels are chosen to represent various physical characteristics of common foods given to dysphagia patients across all age groups, clinical settings, and cultural contexts [20]. Correspondingly, the texture modification of food/liquids is widely used as an intervention strategy for dysphagia, whereas the adoption of thickened beverages and foods with altered textures has been less frequently considered in therapy [23,24]. Thin liquids with rapid flow rates are known to present safety risks for those with dysphagia, since the rapid rate at which the bolus travels from the mouth to the pharynx may not provide the patient with enough time to activate airway protection before the bolus reaches the entrance of the larynx and airway [25,26]. In order to slow the liquid flow and provide the airway with a longer period to close, thicker fluids are often advised [25,27].

On the other hand, particularly thick liquids and solid food contents may demand more force from the tongue to push the content through the oropharynx. Residues might remain in the pharynx following a swallow when an individual has weaker tongue muscles or pharyngeal muscles [25,28,29]. Similarly, solid foods that require chewing might be difficult for individuals with dental problems or poor masticatory muscles. Therefore, the foods are prepared to be easily absorbed or swallowed. Thus, the texture categorization and food/liquid preparation methods highlighted in the IDDSI framework are also represented in CAPS.

The dry (saliva) swallowing and non-swallowing tasks are drawn from our previous review synthesis [19]. Dry (saliva) swallowing identification featured in the majority of wearable systems that assessed swallowing [19]. For non-swallowing, Skowronski et al. [30] proposed a non-swallowing protocol with tasks including yawning, sniffing, tongue movement, humming, throat clearing, coughing, and speech, while Fukuike et al. [22] added gargling, sighing, and sipping tea. Talking, reading, and speech have not been standardized in previous studies, though they might be examined by machine learning. In our protocol, we regulate these skills by including a task pronouncing vowels, which typifies reading/speech and has been used to train phonation biomarkers of dysphagia in machine learning models [31].

On the other hand, traditional non-instrumental bedside screening could be complemented by and embraced together with CAPS. Oral feeding tests are performed by scrutinizing the swallowing process in the oral and pharyngeal phases when consuming liquids, semi-liquids, semi-solids, and solids [11]. They could be well-integrated with CAPS and apply the IDDSI framework. The water swallowing test (or the 3 oz water swallowing test) evaluates aspiration risks in dysphagia by watching out for coughing, choking, and voice changes [32]. The pre-test phase might achieve some goals of the water swallowing test by observing these acts when the patient attempts a larger bolus. The modified Mann Assessment of Swallowing Ability (mMASA) considers several items related to non-swallowing events, such as saliva control, cough reflex, and hypernasality upon sustaining a pronunciation of “AH” [33].

There are several limitations to the protocol. First, the pre-test phase urges the participants to intake as large a bolus as they can, which imposes risks of choking and should be strictly monitored by clinicians. Secondly, the time required for the protocol is relatively long, and the participants consume an amount of food/liquids that could affect their perception and performance due to feeling full. Thirdly, the protocol involves a series of tasks that may be difficult for some individuals, such as those with dementia or mild cognitive decline, in order to comply with the steps or give correct responses (e.g., to determine if they are comfortable to take a larger bolus). We also anticipated that some older adults might refuse to comply because they do not like a particular food, necessitating a switch to other foods/liquids with the same level of texture or thickness. Future studies could consider integrating the continuous or frequent computer-aided swallowing assessment with dysphagia training for those who are at risk, for example, using virtual reality with biofeedback systems [34]. In the long run, we aim to establish a smart dementia care home by integrating various continuous monitoring systems for dementia-related issues, including dysphagia [19], wandering [35] and agitation [36,37], and balance training [38], through the Internet of Medical Things (IoMT) [39,40].

## 4. Conclusions

This paper proposes the Comprehensive Assessment Protocol for Swallowing (CAPS) based on our existing review, protocols from existing papers, and existing standards, including the IDDSI framework and water swallowing test, etc. The protocol consists of a pre-test phase to determine the bolus volume and an assessment phase based on dry (saliva) swallowing, wet swallowing, and non-swallowing tasks. The protocol is designed for the development of computer-aided screening tool, in which the swallowing maneuvers can be classified using a machine learning model or other classifier algorithms. Nevertheless, the protocol design is generic, and we anticipate that it could also be applied to other non-instrumented bedside screening methods.

## Figures and Tables

**Table 1:** Bolus intake for wet swallowing in the pre-test phase.

Intake	IDDSI Level	Step	Food Texture and Drink Thickness	Examples
Liquids	Level 0	1	Thin drink	Water, milk, tea
	Level 1	2	Slightly thick drink	Anti-regurgitation (AR) infant formulas
	Level 2	3	Mildly thick drink	Milkshakes, thick shakes
	Level 3	4	Moderately thick drink or liquidized food	Smooth yoghurt, fruit juice, liquidized poultry, fish, or vegetables
Foods/Transitional Foods	Level 4	5	Extremely thick drink or pureed food	Pureed mince and gravy, smooth mashed potatoes, pureed vegetables, thick cream
	Level 5	6	Minced and moist food	Mashed fruit or vegetables, scrambled egg, milk pudding
	Level 6	7	Soft and bite-sized food	Steamed or boiled vegetables, cooked fish, tender meat less than 1.5 cm × 1.5 cm
	Level 7	8	Easy to chew or regular food	Steamed or boiled vegetables, cooked fish, tender meat with no specific size requirements

MIDDSI: International Dysphagia Diet Standardization Initiative. Information acquired from © The International Dysphagia Diet Standardization Initiative 2019, @ https://iddsi.org/framework/Licensed (accessed on 5 February 2023) under the CreativeCommons Attribution Sharealike 4.0 License: https://creativecommons.org/licenses/by-sa/4.0/legalcode (accessed on 5 February 2023). Derivative works extending beyond language translation are NOT PERMITTED.

**Table 2:** The six maneuvers for the non-swallowing tasks.

Step	Maneuvers	Instructions
1	Throat clearing	Participants should make the sound “ahem” by inhaling slightly and then exhaling more forcibly.
2	Yawning	Participants should open their jaw widely, take in a deep breath, and then quickly exhale.
3	Sniffing	Participants should draw air into their nose in short breaths.
4	Coughing	Participants should take a deep breath, hold it for 2–3 s, and use their stomach muscles to forcefully expel the air.
5	Humming	Participants should hold their lips together and sing the alphabet song.
6	Pronouncing vowels	Pronouncing vowels: participants should slowly read out the vowels “\a\”, “\e\”, “\i\”, “\o\”, “\u\” and sustain for 3 s.

## Data Availability

Not applicable.
